# Antibacterial, antibiofilm and cytotoxic activities of *Terminalia fagifolia* Mart. extract and fractions

**DOI:** 10.1186/s12941-015-0084-2

**Published:** 2015-04-19

**Authors:** Alyne Rodrigues de Araujo, Patrick Veras Quelemes, Márcia Luana Gomes Perfeito, Luíza Ianny de Lima, Melka Coêlho Sá, Paulo Humberto Moreira Nunes, Graziella Anselmo Joanitti, Peter Eaton, Maria José dos Santos Soares, José Roberto de Souza de Almeida Leite

**Affiliations:** Center for Research on Medicinal Plants, Federal University of Piauí, Piauí, Brazil; Biodiversity and Biotechnology Research Center, Biotec, Federal University of Piauí, Parnaíba, Piauí Brazil; Federal University of Rio Grande do Norte, Rio Grande do Norte, Brazil; Department of Physiology and Biophysics, Federal University of Piauí, Piauí, Brazil; Campus Ceilândia, University of Brasília, Brasília, Brazil; UCIBIO, REQUIMTE, Departamento de Química e Bioquímica, Faculdade de Ciências, Universidade do Porto, Porto, Portugal; Department of Veterinary Morphology and Physiology, Federal University of Piauí, Piauí, Brazil

**Keywords:** *Terminalia*, *Staphylococcus*, Antibacterial, Antibiofilm, Cytotoxicity

## Abstract

**Background:**

The methicillin resistance of bacteria from the genus *Staphylococcus* and its ability to form biofilms are important factors in pathogenesis of these microorganisms. Thus, the search for new antimicrobials agents, especially from plants, has been intensified. In this context, *Terminalia* species have been the subject of research for many pharmacological activities. In this study we evaluated the antibacterial, antibiofilm and cytotoxic activities of the ethanol extract (EtE) from *Terminalia fagifolia* stem bark as well as that of three fractions of the extract (AqF, HaF and WSF).

**Methods:**

We determined the minimum inhibitory concentration (MIC) by microdilution in 96-well plates, where the strains were exposed to serial dilutions of the ethanol extract and fractions, ranging from 12.5 to 400 μg/mL. We then determined the minimum bactericidal concentration (MBC), seeding the inoculum (10 μL) with concentrations equal to or greater than the MIC in Mueller-Hinton agar. To test the antibiofilm activity biofilm formation was induced in the presence of concentrations equivalent to 1/2, 1/4 and 1/8 of the MIC extract or fraction tested. In addition, the effect of the EtE and the fractions on cell viability was tested by the MTT assay on human MCF-7 breast cancer and mouse fibroblast NIH/3T3. To obtain high-resolution images of the effect of the aqueous fraction on the bacterial morphology, atomic force microscopy (AFM) imaging of treated *S. aureus* cells was performed.

**Results:**

We observed antibacterial activity of EtE and fractions with MICs ranging from 25–200 μg/mL and MBCs ranging from 200–400 μg/mL. Regarding antibiofilm activity, both the EtE as the AqF, HaF and WSF fractions showed significant inhibition of the biofilm formation, with inhibition of biofilms formation of over 80% for some strains. The EtE and fractions showed a moderate cytotoxicity in cell line NIH/3T3 viability and potential antitumoral activity on human breast cancer cell line MCF-7. The microscopic images obtained revealed morphological changes to the *S. aureus* ATCC 29213 surface caused by AqF, as well as significant size alterations.

**Conclusions:**

The results show potential antibacterial, antibiofilm and antitumoral activities of the ethanol extract and fractions of *T. fagifolia*.

## Background

The shortage of new antimicrobial has been characterized as a public health emergency and pathogenic microorganisms resistant to antimicrobial used in therapy of infections have appeared both in the community and in hospitals. Among them, staphylococci stand out because they belong to a diverse group of bacteria that cause diseases ranging from skin infections to bacteremia. The two major opportunistic pathogens of this genus, *Staphylococcus aureus* and *Staphylococcus epidermidis*, colonize a considerable portion of the human population [1-3].

*Staphylococcus aureus* can cause a range of serious infections, with rates of morbidity and mortality of up to 64%, this pathogenicity reflects its ability to produce a variety of toxins, and to firmly adhere to prosthetic materials, apart from the capacity to develop resistance to antimicrobial agents [4]. *S. epidermidis*, despite being an important commensal, has emerged as the most significant pathogen related to the implantation of medical devices infections [5-7].

Resistance to methicillin is an additional important factor in the establishment of infections caused by *S. aureus* or by *S. epidermidis* and it is becoming more and more prevalent. This resistance profile, along with the ability to form biofilm complicates the treatment of infections caused by these pathogens [8-11].

After the establishment of the biofilm, the diffusion of antibiotics is hampered, the inner layers bacteria begin to have a low metabolic rate, physiological changes occur in the growth mode, among other mechanisms of resistance [12]. New strategies are needed to remove these infections mediated by the biofilm. In this context a renewed interest in natural substances has drawn attention to plants rich in secondary metabolites, known for their antimicrobial properties [13].

The *Terminalia* genus, belonging to the Combretaceae family, comprising around 200 species widely used in folk medicine, *Terminalia fagifolia* Mart. is found in the Brazilian Cerrado and known popularly as “*capitão, capitão-do-cerrado, capitão-do-campo* and *mirindiba*” [14].

From the stem bark of *T. fagifolia* two 1,3-diarylpropanes, seven flavanones, two chalcones, one flavan, nine triterpenes, gallic acid and sitosterol have been characterized by Nuclear Magnetic Resonance (NMR) [15]. Previous studies on ethanol extracts of the *T. fagifolia* Mart. leaves have also revealed the presence of several specific chemical compounds, such as (+)-catechin, sitosterol-3-*O*-β-D-glucopyranoside, α- and β-tocopherol, a mixture of lupeol, α- and β-amyrin, and a mixture of glycosides flavonoids [16]. These compounds exhibit potential antibacterial activity among several other pharmacological activities [15,16].

Therefore, this study aimed to test the antibiofilm activity the ethanol extract and aqueous, hydroalcoholic and water-soluble fractions of the stem bark of the *Terminalia fagifolia* Mart. (Combretaceae) against sensitive and resistant bacteria of the genus *Staphylococcus* sp.

## Materials and methods

### Plant material

The stem bark of *T. fagifolia* were collected in November 2006 in the city of Timon, Maranhão State, Brazil. The species was identified by Dr. Gardene Maria de Souza of the Graziela Barroso Herbarium, Federal University of Piauí, where a specimen was deposited under TEPB number 21691.

### Extraction

The stem bark of the *T. fagifolia* was air dried, crushed and subjected to a maceration process six times with ethanol (1:1) at room temperature. After removal of the solvent on a rotary evaporator (55°C under reduced pressure) followed by lyophilization, ethanol extract (EtE) was obtained. A portion of the extract was separated for biological testing and the remaining material was suspended in 1,200 mL of a mixture of H_2_O/MeOH (2:1) and partitioned with ethyl acetate. The organic phase was concentrated, then suspended in MeOH/H_2_O (9:1), and finally extracted with hexane, thus providing the following fractions: aqueous (AqF), hydroalcoholic (HaF) and hexane (not used in this study), in addition to the water-soluble precipitate (WSF) [17].

### Bacterial strains and inoculum standardization

Ethanol extract (EtE) and its aqueous (AqF), hydroalcoholic (HaF) and water-soluble (WSF) fractions were tested against *Staphylococcus aureus* (ATCC 29213), *S. aureus* COL (MRSA, Methicillin Resistant *Staphylococcus aureus*), *S. aureus* WB69 (MRSA), *Staphylococcus epidermidis* (ATCC 12228), *S. epidermidis* H111 (MRSE - Methicillin Resistant *Staphylococcus epidermidis*) and *S. epidermidis* 70D (MRSE). Bacteria were spread over Mueller-Hinton agar and aerobically incubated for 24 h at 37°C, after which they were collected and suspended in sterile saline [0.85% NaCl (w/v)] to reach an absorbance between 0.08 and 0.10, at 625 nm was obtained, representing approximately 1–2 × 10^8^ CFU/mL. This bacterium solution was diluted 1:10 and used for the following procedures.

### Antibacterial activity

Minimum inhibitory concentration (MIC) determinations were performed through microdilution of the Mueller–Hinton broth following the recommendations of the CLSI [18]. Thus 8 μL of each extract or fraction were added to microtiter wells containing 187 μL Mueller-Hinton broth followed by 2-fold serial dilutions with final concentrations ranging from 12.5 - 400 μg/mL.

The standardized inoculum (5 μL) was added to give a final concentration of 5 × 10^5^ CFU/mL, reaching a final volume of 100 μL in each well. In addition, Oxacilin (for sensitive strains) and Vancomicin (for MRSA and MRSE) were included in other microplate. Bacterial growth control consisted of 95 μL of culture medium and 5 μL of bacteria inoculums added to three wells. DMSO was used as a negative control. After incubation at 37°C for 24 h, MIC was defined as the lowest concentration of extract at which no visible growth could be detected. The results were enhanced by the use of 10 μL of Triphenyl Tetrazolium Chloride 1% (TTC) (Sigma, St. Louis, MO, USA), which may be reduced when there is bacterial growth, forming the triphenyl formazan with red color.

In order to determine the minimal bactericidal concentration (MBC), 10 μl of the wells that showed results equal to or greater than the MIC were seeded onto Mueller-Hinton Agar (MHA), before TTC addition. After 24 h of incubation at 37°C, the MBC was considered the lowest concentration that inhibited visible bacterial growth on the agar. All the tests were performed in triplicate.

### Antibiofilm activity

The assay was performed in 96 well microtiter plates, to each well we added the bacterial suspension (5×10^5^ CFU/mL), the ethanol extract and its fractions at concentrations less than MIC (1/2, 1/4 and 1/8 of the MIC) and TSB medium (Tryptical Soya Broth) which was supplemented with 0.5% glucose, then the microplate was incubated at 37°C for 24 h under aerobic conditions. After this incubation period, the culture medium was removed and the wells washed twice with distilled water. Then, the biofilm was fixed with 100 μL of Methanol (PA) for 15 minutes, stained with crystal violet 0.1% (v/v) and rinsed with water. Biofilm formation was evaluated by adding 100 μL of 95% ethanol to the wells, and the plates subjected to spectrophotometric reading at 630 nm, according to Stepanovic et al. [19] with modifications. The results were expressed as percent inhibition of biofilm formation, considering the absorbance of the positive control (wells to which no potential antibacterial agents were added) as 0% inhibition. All tests were made in quadruplicate (four replicate wells).

### Cell culture

The human breast cancer cell line MCF-7 was purchased from American Type Culture Collection (ATCC) and murine fibroblast cell line NIH/3T3 was purchased from the Cell Bank of the Federal University of Rio de Janeiro (Rio de Janeiro, Brazil). The cells were routinely maintained in culture flasks (TPP, Switzerland), at 37°C in 5% CO_2_, in DMEM media with 100 IU/mL penicillin and 100 μg/mL streptomycin supplemented with 10% (v/v) heat inactivated fetal bovine serum (Life Technologies, USA).

### MTT assay and cytotoxicity analysis

For determining the cytotoxic activity, cells were seeded on 96-well plates at a density of 5x10^3^ cells per well in culture medium overnight at 37°C in in 5% CO_2_. The medium was changed and cells were incubated with different concentrations of EtE, AqF, HaF, WSF of the *T. fagifolia* trunk bark (12.5 – 400 μg/mL) prepared from a stock solution diluted in Dimethyl Sulfoxide (DMSO). As control groups, cells were treated with DMSO at the same concentration used in the treatments and cells treated with normal media (100% cell viability). The final concentration of DMSO in each well was 1% (v/v) and the treatments were conducted in triplicate for each experimental group.

Cell viability was determined by a 3,4,5-dimethylthiazol-2,5 biphenyl tetrazolium bromide (MTT – Life Technologies, USA) assay. After 24 h treatment, 15 μL of MTT solution (5 mg/mL in PBS) were added to each well. After 2 h of incubation at 37°C in 5% CO_2_, the culture media was aspirated and 100 μL of DMSO were added. The absorbance was monitored using a spectrophotometer with a microplate reader at a wavelength of 595 nm (SpectraMax; Molecular Devices, Sunnyvale, California, USA).

### Atomic Force Microscopy (AFM)

To perform Atomic Force Microscopy (AFM), the MIC value of aqueous fraction (AqF) against *S. aureus* ATCC 29213 was determined again as described before, using inocula of 1–2 × 10^8^ CFU/mL with AqF concentration ranging from 50 – 800 μg/mL. After incubation for 24 hours, 30 μL of the culture medium containing the treated (at MIC value) or non-treated bacteria were deposited onto a clean glass surface followed by drying in bacteriological incubator at 35°C for 10 min. The samples were then gently rinsed with deionized water to remove salt crystals, and other unwanted growth medium components, and dried again under the same conditions, before AFM analysis. All samples were prepared at the same time, exposed to the same conditions and examined within 8 hours of deposition [20]. AFM was carried out with a TT-AFM microscope from AFM Workshop (USA). The analysis of the effect of AqF on *S. aureus* cells was carried out in vibrating mode, using cantilevers (NSG10/NT-MDT) with resonant frequency approximately 240 kHz. Images were analyzed using Gwyddion software version 2.40. Multiple areas of each sample were examined and selected representative images from images obtained of the treated and untreated bacteria are shown.

### Statistical analysis

Statistical analysis of the results obtained was done with the GraphPad Prism ® 5.0 software (GraphPad Sofware Inc.) for the antibiofilm assay and cell viability assays for which the analysis of variance (ANOVA) followed by post-test Tukey or post-test Bonferroni to assess differences between the groups was carried out. The results were expressed as the mean ± SEM (Standard Error of Mean). To compare the size of the bacteria obtained by AFM, *T*-test was applied and the results were expressed as the mean ± SD. A *p* < 0.05 was considered statistically significant.

## Results and discussion

The results of the minimum inhibitory concentration (MIC) test showed that the ethanol extract (EtE) and aqueous (AqF), hydroalcoholic (HaF) and water soluble (WSF) fractions effectively inhibited the growth of all strains of *S. aureus* and *S. epidermidis* evaluated. The MICs ranged from 25–200 μg/ml, thereby demonstrating the potential of these substances as antibacterial agents, in Table 1 we can see that in general, *S. epidermis* was inhibited at lower concentrations by the EtE and fractions than *S. aureus*. In summary, the AqF was the most effective, inhibiting all strains tested at 25 to 50 μg/mL, the EtE was intermediate, being quite effective against the *S. epidermis* strains, but less so against *S. aureus* strains, and the HaF was the least effective.

**Table 1:** **Minimum Inhibitory Concentration and Minimum Bactericidal Concentration (MIC and MBC) of EtE, HaF, AqF and WSF in g/mL**

**Bacterial strains**	**Ethanol extract**		**Hydroalcoholic fraction**		**Aqueous fraction**		**Water soluble fraction**	
	**MIC**	**MBC**	**MIC**	**MBC**	**MIC**	**MBC**	**MIC**	**MBC**
*S. aureus* ATCC 29213	100	400	200	200	50	200	50	200
*S. aureus* COL	100	>400	200	>400	50	>400	100	>400
*S. aureus* WB69	50	>400	100	200	50	400	50	400
*S. epidermidis* ATCC 12228	25	400	50	>400	25	400	25	>400
*S. epidermidis* 70D	25	>400	25	>400	25	>400	25	>400
*S. epidermidis* H111	25	>400	25	>400	25	400	25	>400

After determining the MIC, the minimum bactericidal concentration (MBC) was determined and it was only found in some bacteria at the evaluated concentrations. The AqF had an MBC equal to 400 μg/mL against *S. aureus* WB69 and *S. epidermidis* H111 (Methicillin-Resistant clinical specimens). The lowest MBC found (200 μg/mL) was for HaF against *S. aureus* WB69 and *S. aureus* ATCC 29213. For the *S. aureus* COL and *S. epidermidis* 70D strains, the extract or fractions showed no MBC at the concentrations tested (Table 1).

The indiscriminate use of antibiotics by the population is one of the causes for the increasing resistance of microorganisms that result in a reduction of available therapeutic alternatives. This factor coupled with the high cost of production of synthetic compounds prompts further research for new antimicrobials that are effective against pathogens resistant to currently used drugs [21].

Herbal medicines may be an alternative because they present secondary metabolites that are active against a wide range of microorganisms. Extracts derived from other plants of the genus *Terminalia* that have been studied elsewhere also showed antibacterial effect, but with lower activity than the results presented above [21,22]. For instance Bag et al. [23] studying the effect of the ethanol extract of *Terminalia chebula* found MIC of 975 μg/mL against *S. aureus*.

The MIC of an agent for a given microorganism is the lowest concentration of the agent required to inhibit growth of a bacterial inoculum in a standardized test, while the MBC is the lowest concentration that an agent is able to kill bacteria present in an inoculum. An agent is generally considered bactericidal if the MBC is less than or equal to four times the MIC value [24]. Thus, the *T. fagifolia* EtE and fractions thereof may be considered bactericidal to *S. aureus* ATCC 29213, as well as the HaF for *S. aureus* WB69.

The antibacterial effect shown by the EtE and its fractions may be due to the chemical composition of the extract, which is rich in flavonoids (flavanones, chalcones and flavanes), 1,3-diarilpropanos, pentacyclic triterpenes glycosylated and non-glycosylated [15]. In addition, Nunes et al. [17] suggested the presence of polar compounds with antimicrobial potential, like flavonoids, glycosylated flavonoids, and saponins in the AqF and HaF fractions.

Studies describe the action of naturally occurring molecules as antimicrobial agents such as terpenoids, glucocorticoids, flavonoids and polyphenols that are small molecules produced by plants and are able to inhibit many bacterial species, particularly Gram-positive microorganisms. In some cases these microorganisms are resistant to disinfection, particularly when they form biofilms [25].

With the exception of *S. epidermidis* ATCC 12228, all the bacterial strains tested in the previous section are capable of forming biofilms. Thus, we further examined the antibiofilm activity of EtE and its partitions (AqF, HaF and WSF). Both the EtE and the AqF, HaF and WSF fractions showed inhibition of biofilms, especially at the highest concentration used. It is noteworthy to observe that the tested concentrations were equivalent to 1/8, 1/4 and 1/2 the MIC of each substance in relation to the different strains tested, and that they ranged from 100–3.12 μg/ml.

**Figure 1:**
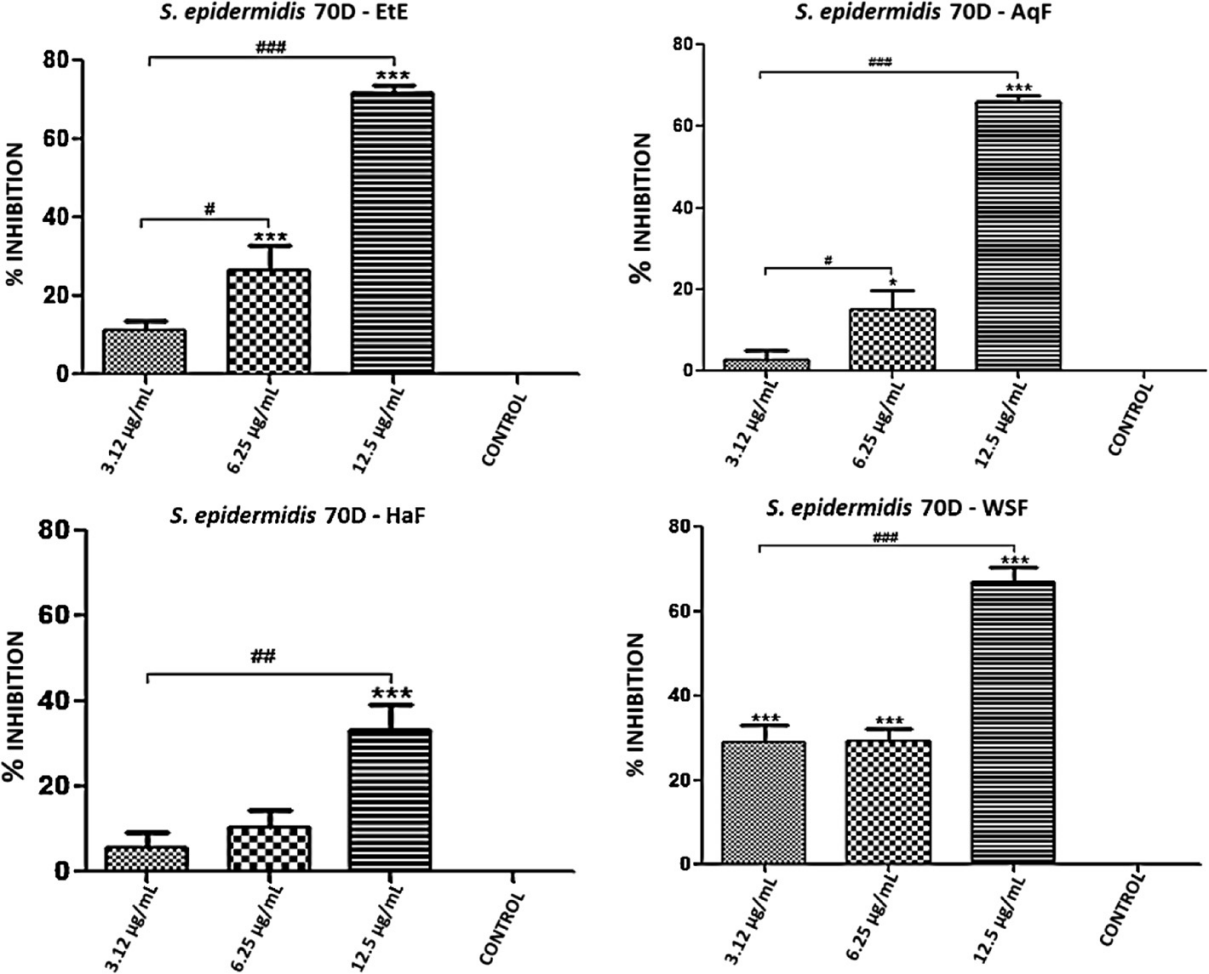
Percent inhibition of biofilm formation exhibited by ethanol extract (EtE) and fractions (AqF, HaF and WSF) of *T. fagifolia* against *S. epidermidis* 70D biofilm.

**Figure 2:**
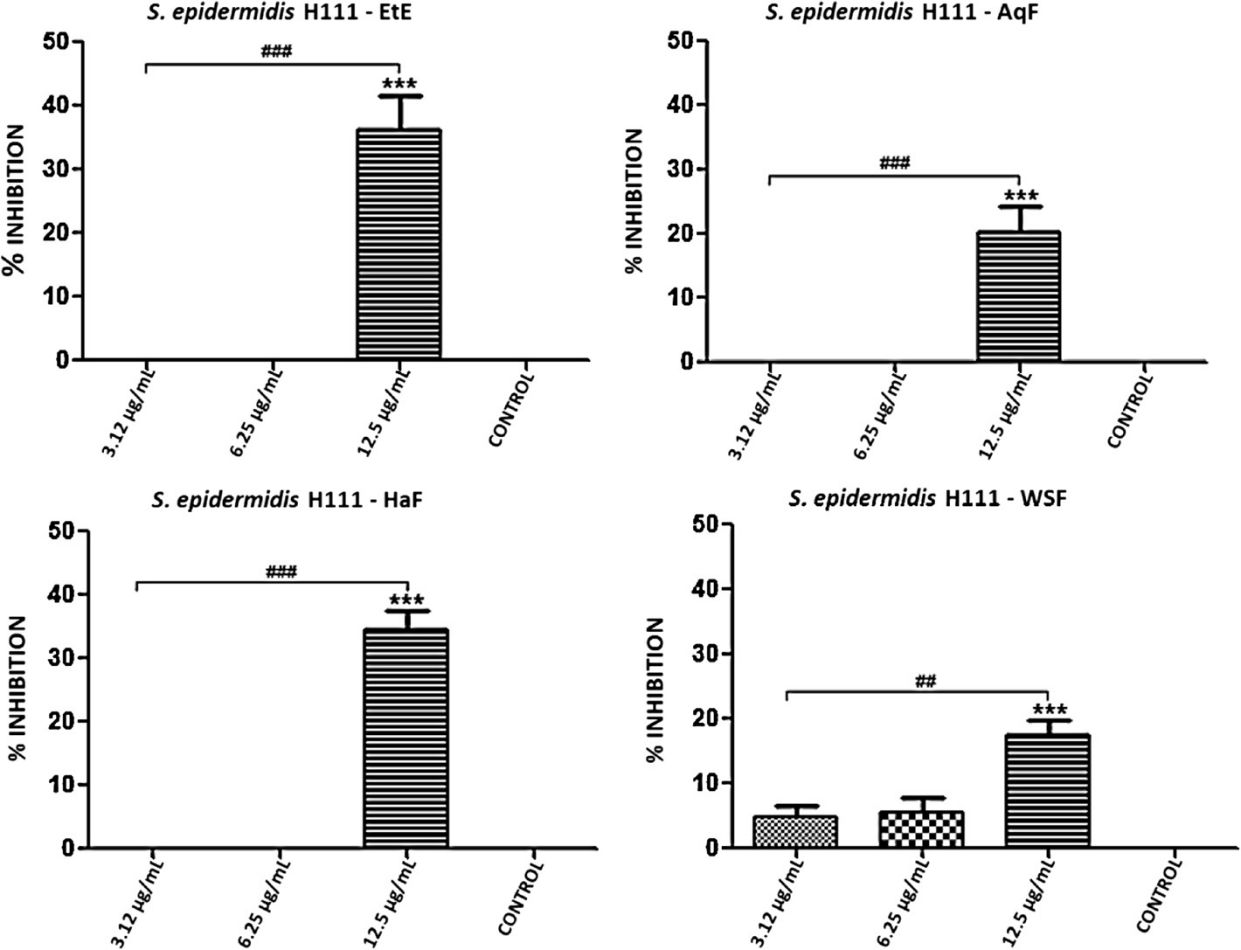
Percent inhibition of biofilm formation exhibited by ethanol extract (EtE) and fractions (AqF, HaF and WSF) of *T. fagifolia* against *S. epidermidis* H111 biofilm.

In regards to *S. epidermidis* 70D (MRSE), only the WSF showed antibiofilm effect at the three tested concentrations (12.5, 6.25 and 3.12 μg/ml). The EtE and AqF showed antibiofilm activity at the two highest concentrations used (6.25 and 12.5 μg/ml) for that strain, unlike the HaF which exhibited such activity only at the highest concentration (12.5 μg/ml), as illustrated in Figure 1. For *S. epidermidis* H111 (MRSE), only the highest concentration tested (12.5 μg/ml) and the EtE fractions (AqF, HaF and WSF) showed statistically significant inhibition of biofilm formation (Figure 2).

**Figure 3:**
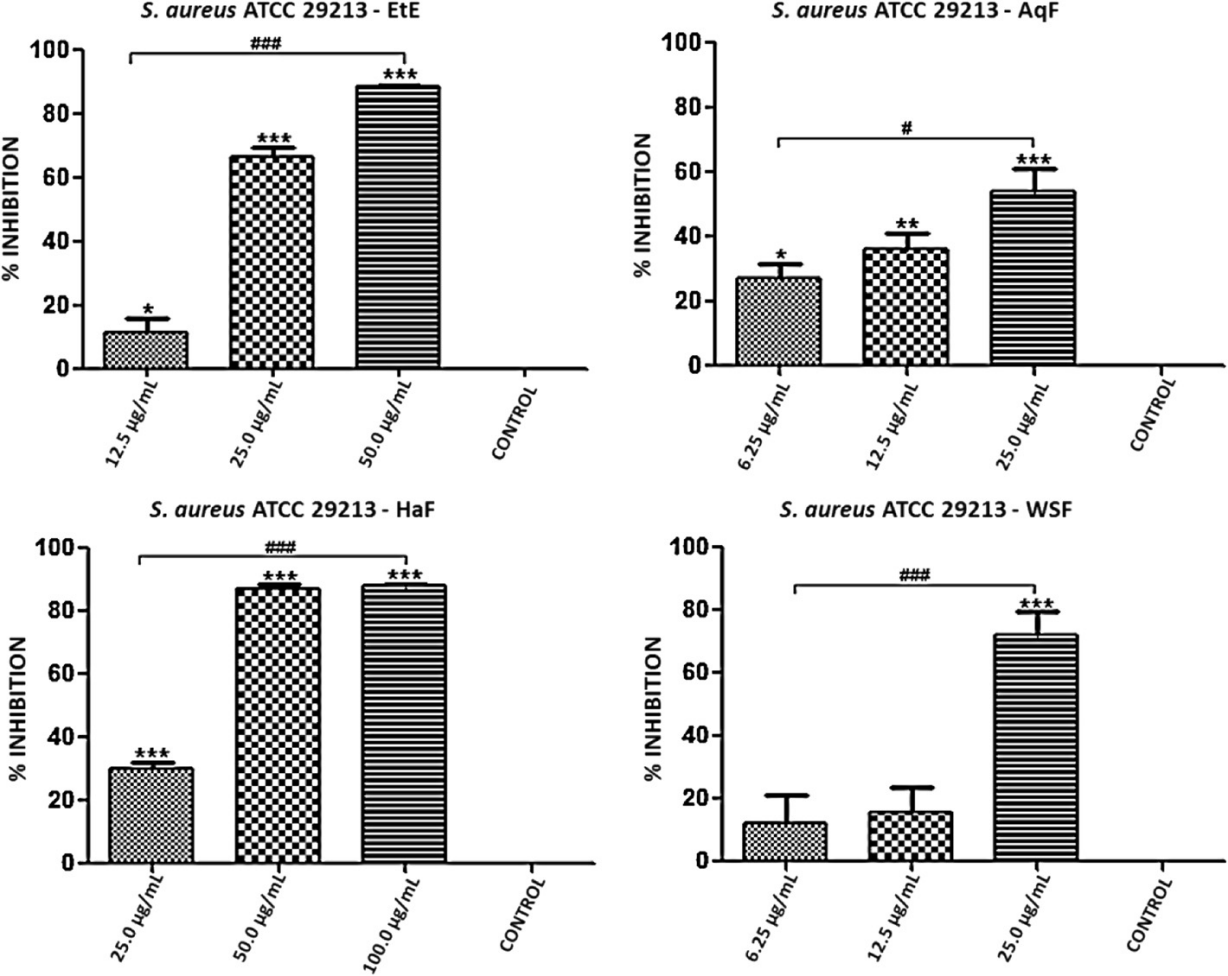
Percent inhibition of biofilm formation exhibited by ethanol extract (EtE) and fractions (AqF, HaF and WSF) of *T. fagifolia* against *S. aureus* ATCC 29213 biofilm.

**Figure 4:**
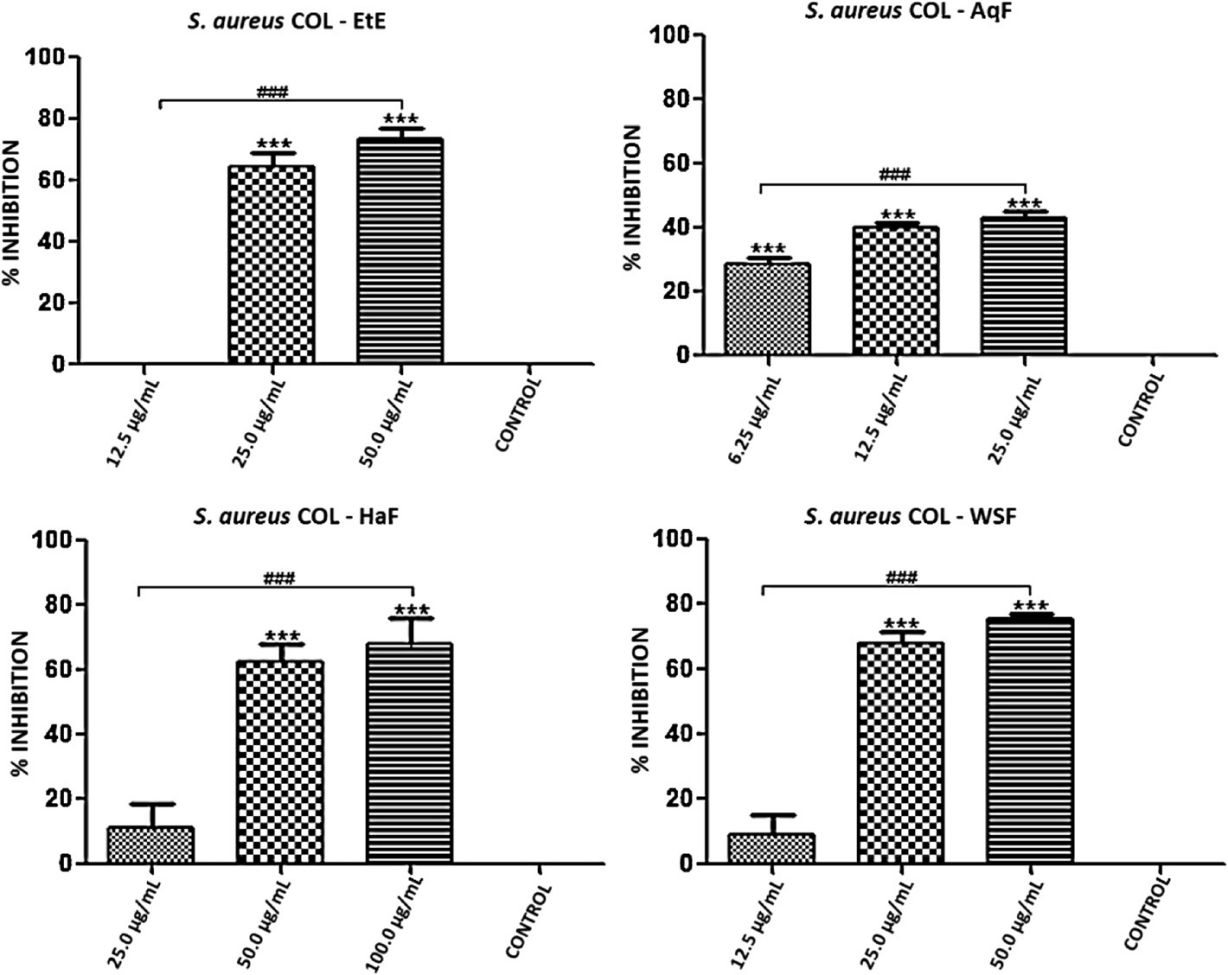
Percent inhibition of biofilm formation exhibited by ethanol extract (EtE) and fractions (AqF, HaF and WSF) of *T. fagifolia* against *S. aureus* COL biofilm.

**Figure 5:**
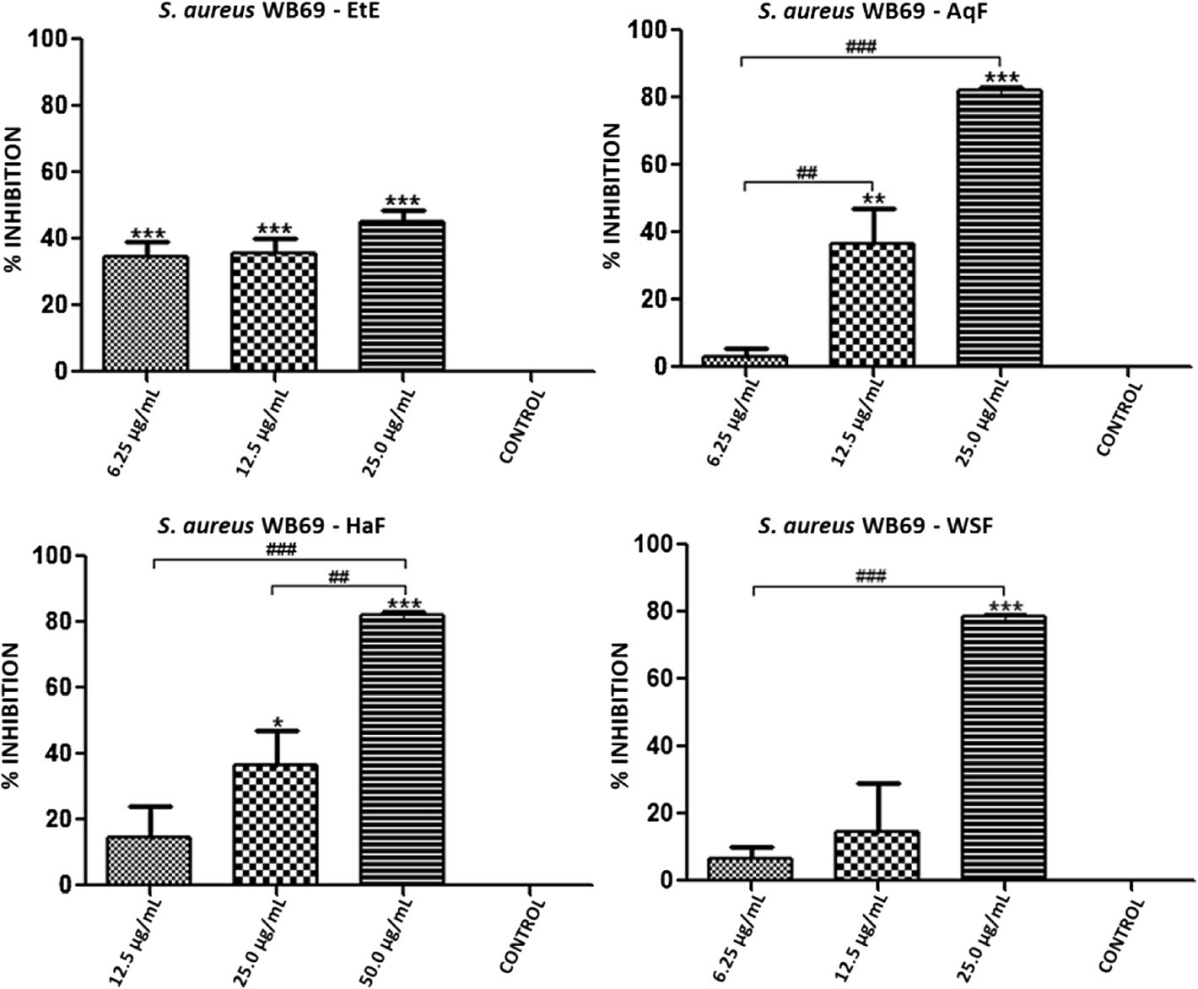
Percent inhibition of biofilm formation exhibited by ethanol extract (EtE) and fractions (AqF, HaF and WSF) of *T. fagifolia* against *S. aureus* WB69 biofilm.

The biofilm of *S. aureus* ATCC 29213 was inhibited by EtE, HaF and AqF at all concentrations used. The WSF showed inhibition only at the highest concentration. Furthermore AqF showed a concentration dependent inhibition at the lower concentrations tested for this strain, 25, 12.5 and 6.25 μg/ml (Figure 3). In regards to the *S. aureus* COL (MRSA) strain, only AqF showed inhibitory effect at the lower concentration (6.25 μg/mL), as it can be seen in Figure 4. Only the EtE was able to inhibit *S. aureus* WB69 biofilm at the three concentrations tested (Figure 5).

The formation of biofilms, highly organized bacterial complexes, is increasingly recognized as an important virulence factor for *Staphylococcus* sp [12]. The antimicrobial agents derived from natural products may represent a suitable alternative to limit this process.

Studies of antibiofilm activity derived from plants of the Combretaceae family are scarce. The antibiofilm effect of extracts from various plants of the Brazilian caatinga has been evaluated, among them one belonging to Combretaceae, *Buchenavia tetraphylla* (Aubl.). However, this species showed no antibiofilm activity [26].

In order to have cell adhesion onto the surface, the bacteria synthesize and release exopolysaccharides, which are considered key components in determining the structure and functional integrity of biofilm, besides acting as adhesives and defensive barrier, protecting the cells from being detached by the flow of substances. Studies report that adherence of *Staphylococcus* sp. to polypropylene films probably occurs through hydrophobic bonds such as Van der Waals interactions therefore depending on the electrical charge and surface hydrophobicity of materials and organisms [27,28].

Thus unknown metabolic changes may be involved in the inhibition of biofilm formation promoted by EtE and the fractions AqF, HaF and WSF. Among the possible changes one can cite a probable decrease in the production and release of exopolysaccharides, and a change in electrical charge and/or hydrophobicity of the bacterial membrane. To confirm the mechanism by which EtE and fractions act, more specific protocols need to be performed.

**Figure 6:**
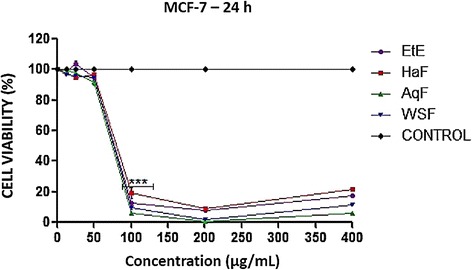
Effect of ethanol Extract (EtE) and fractions (AqF, HaF and WSF) of *T. fagifolia* on cell viability of cells of breast cancer - MCF7 within 24 hours.

**Figure 7:**
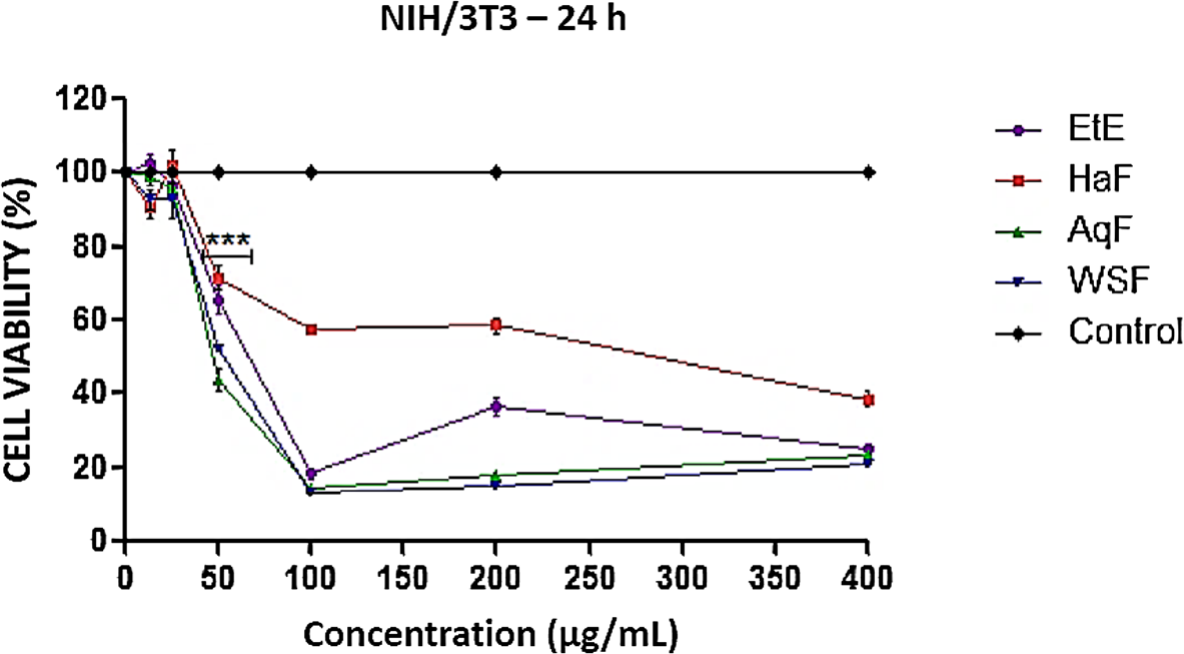
Effect of ethanol extract (EtE) and fractions (AqF, HaF and WSF) of *T. fagifolia* on cell viability of NIH/3T3 murine fibroblasts within 24 hours.

To verify the cytotoxic effect of ethanol extract and fractions, we sought to evaluate its effect on cell viability of tumor cells MCF-7 and non- tumorigenic NIH-3T3. The cytotoxicity of *T. fagifolia* ethanol extract and fractions demonstrated marked cytotoxicity to tumor cells, 100% of decreasing cell viability at concentrations ≥ 100 μg/mL, as well as a reduction in cell viability of non-tumorigenic cells NIH-3T3, mainly starting at the concentration of 50 μg/mL, as shown respectively in Figures 6 and 7.

Derivatives of plants of the *Terminalia* genus showed cytotoxic activity in cancer cell lines by inducing apoptosis and against strains of mouse fibroblast NIH-3T3 with reduction in cell viability, such as the results found in this research [29-31]. However in another study the acute oral toxicity of EtE and fractions was evaluated in mice and the authors established an limit oral dose of 2000 mg/kg in male and female mice. Therefore this extract and fractions could be classified as of low acute toxicity hazard category 5 according to the United Nations Globally Harmonized System of Classification and Labeling of Chemicals [17].

**Figure 8:**
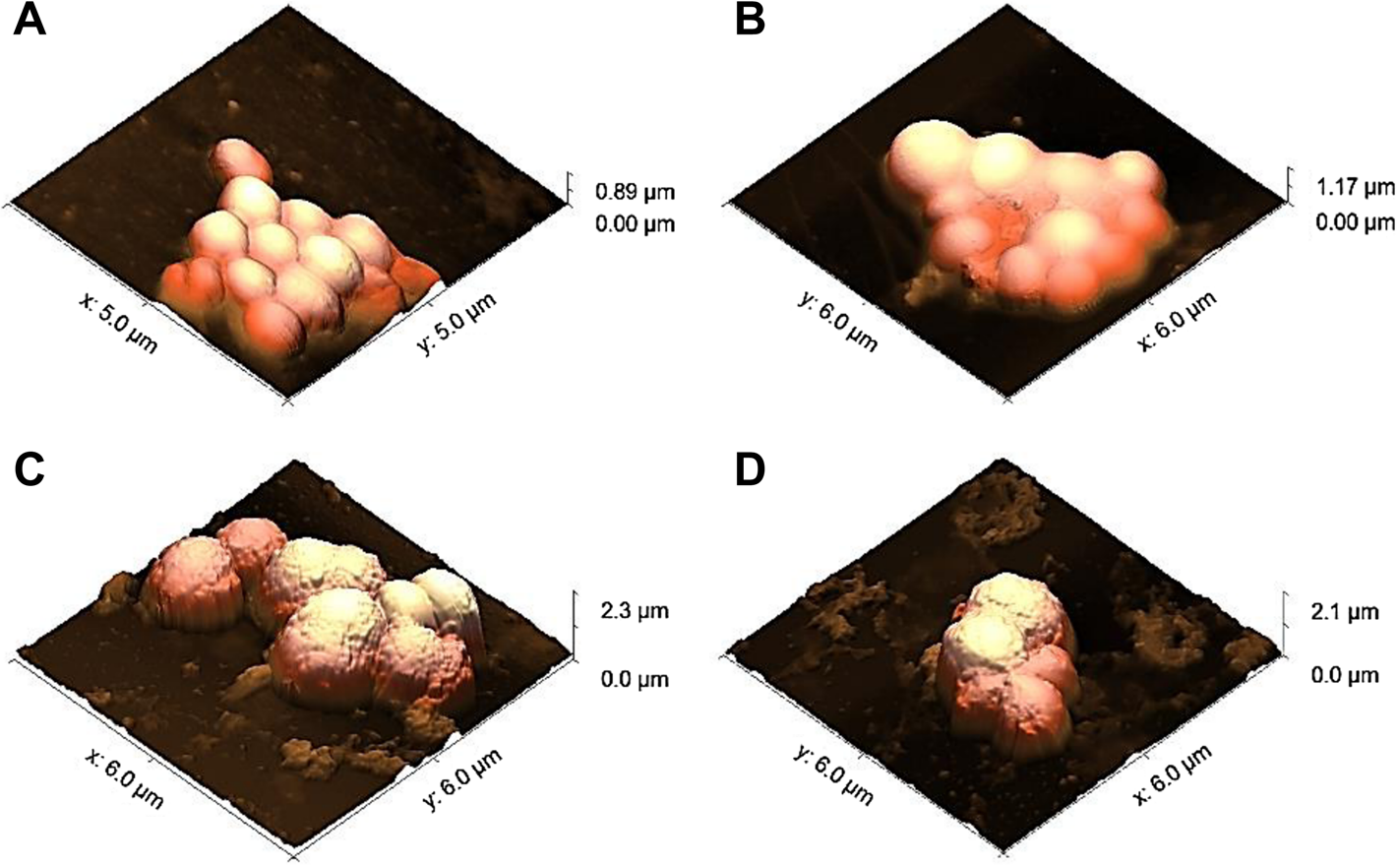
AFM height images showing *S.aureus* ATCC 29213 before **(A and B)** and after **(C and D)** of exposure to the aqueous fraction (AqF) of the ethanol extract of *T. fagifolia*.

**Figure 9:**
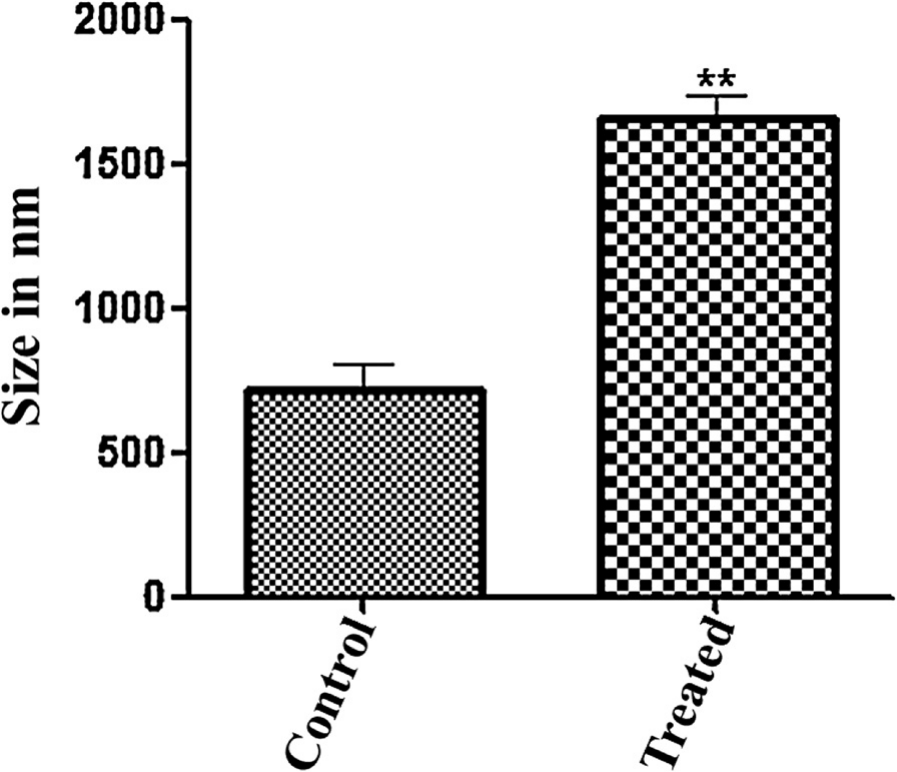
Mean size of treated and untreated (control group) *S. aureus* ATCC 29213 obtained by AFM.

The morphological effect of AqF of the ethanol extract of *T. fagifolia* on *S. aureus* ATCC 29213 cells is shown in Figure 8, we performed atomic force microscopy on only this selected sample, since it was shown (see above) to be the most effective in bacterial growth. Figure 8A and B show the morphology of non-treated bacteria, while Figure 8C and D show changes in the appearance in the cell envelope of treated bacteria. It was found that the treated bacteria had increased roughness on the cell surface compared to the smooth surfaces of the non-treated bacteria, and furthermore we see a significant increase in the size of bacteria treated, observe the Z scales of Figure 8 panels A and B, compared with the panels C and D. This effect was observed in all the cells imaged by AFM, and the overall average of all cell heights are plotted in Figure 9. These increase of the size observed in the Figure 9 may be due to alterations in membrane integrity caused by AqF, which may lead to changes in cell osmolarity without the occurrence of lysis in the observed images. The fact that we can observed cell wall changes, and even cellular size alteration without gross shape alteration or cell collapse could be attributed to the fact that the Gram positive cell wall is mechanically rather strong compared to Gram-negative organisms.

## Conclusions

The ethanol extract and fractions showed great antibacterial activity against all tested strains, and in many cases, we also observed antibiofilm activity, with biofilm formation inhibition over 80% for some strains. As the extracts showed moderate cytotoxicity for the NIH/3T3 cell lines and potential antitumoral activity, confirming pharmacological potential of extracts from the *T. fagifolia* species, which is a common plant in the Brazilian Cerrado biome and widely used in folk medicine. The AFM images obtained revealed morphological changes to the *S.* aureus ATCC 29213 cell surface caused by AqF, as well as significant size alterations.

## Abbreviations

EtE: Ethanol extract from the *Terminalia fagifolia*
AqF: Aqueous fraction of the ethanol extract from the *Terminalia fagifolia*
HaF: Hydroalcoholic fraction of the ethanol extract from the *Terminalia fagifolia*
WSF: Water soluble fraction of the ethanol extract from the *Terminalia fagifolia*
